# High-resolution melting (HRM) curve analysis as a potential tool for the identification of earthworm species and haplotypes

**DOI:** 10.7717/peerj.13661

**Published:** 2022-06-28

**Authors:** Anna Vaupel, Bernd Hommel, Lukas Beule

**Affiliations:** Julius Kühn Institute (JKI)–Federal Research Centre for Cultivated Plants, Institute for Ecological Chemistry, Plant Analysis and Stored Product Protection, Berlin, Germany

**Keywords:** High-resolution melting (HRM) curve analysis, Earthworm identification, Earthworm taxonomy, COI, Real-time PCR, Species, Haplotypes

## Abstract

**Background:**

Earthworm communities are an important component of soil biodiversity and contribute to a number of ecosystem functions such as soil-nutrient cycling. Taxonomic identification is an essential requirement to assess earthworm biodiversity and functionality. Although morphological identification of species is labour-intensive, it is the most commonly used method due to a lack of cost-efficient alternatives. Molecular approaches to identify earthworms at species and haplotype level such as DNA barcoding are gaining popularity in science but are rarely applied in practice. In contrast to barcoding, the differentiation of PCR products based on their thermal denaturation properties using high-resolution melting (HRM) curve analysis is a fast and cost-efficient molecular closed-tube, post-PCR tool that allows identification of taxa.

**Methods:**

We developed a HRM curve assay to identify eight earthworm species common to agricultural soils in Central Europe (*Allolobophora chlorotica*, *Aporrectodea caliginosa*, *Apo. limicola*, *Apo. longa*, *Apo. rosea*, *Lumbricus castaneus*, *L. rubellus*, and *L. terrestris*). For this, a new primer pair targeting a 158-bp long subregion of the cytochrome c oxidase I (COI) gene was designed. Our HRM assay was further tested for the differentiation of COI haplotypes using 28 individuals of the earthworm species *Allo. chlorotica*. Furthermore, we developed a novel extraction method for DNA from earthworm tissue that is fast and requires minimal consumables and laboratory equipment.

**Results:**

The developed HRM curve assay allowed identifying all eight earthworm species. Performing the assay on 28 individuals of the earthworm species *Allo. chlorotica* enabled the distinction among different COI haplotypes. Furthermore, we successfully developed a rapid, robust, scalable, and inexpensive method for the extraction of earthworm DNA from fresh or frozen tissue.

**Conclusions:**

HRM curve analysis of COI genes has the potential to identify earthworm species and haplotypes and could complement morphological identification, especially for juvenile or damaged individuals. Our rapid and inexpensive DNA extraction method from earthworm tissue helps to reduce the costs of molecular analyses and thereby promote their application in practice.

## Introduction

Soil meso- and macrofauna is a substantial part of soil biodiversity and contributes to key ecosystem functions such as nutrient cycling (*e.g.*, Reichle, 1977) and digestion of pathogens (*e.g.*, Friberg, Lagerlöf & Rämert, 2005; Sofo, Mininni & Ricciuti, 2020). Earthworms are important members of the soil faunal community and involved in multiple beneficial ecosystem functions. For example, earthworms contribute to the decomposition of organic material (*e.g.*, Cortez, 1998) and interact with beneficial as well as phytopathogenic soil microorganisms (*e.g.*, Doube et al., 1994). Earthworms can also serve as an indicator for soil health (Linden et al., 1994). Abundance, biomass and species diversity are common measures to characterize earthworm populations and their potential functions in the soil ecosystem. According to their ecology, earthworms can also be classified into three different major ecological groups (*i.e.*, anecic, endogeic and epigeic species). Recently, however, the suitability of these widely accepted three groups to reflect the functions of earthworms was questioned. Bottinelli & Capowiez (2021) suggested that the seven categories proposed by Bouché (1972) should be used instead. In addition to functional classification, taxonomic identification is an essential prerequisite for the assessment of earthworm biodiversity.

Identification of species by their morphology using regional identification keys (*e.g.*, Graff, 1953; Stöp-Bowitz, 1969; Bouché, 1972; Ljungström, 1970; Sims & Gerard, 1985) is still the most common technique applied in current earthworm surveys (*e.g.*, Pérès et al., 2011; Tsiafouli et al., 2015; Ashwood et al., 2019). The main advantages of this method are its low costs and few required equipment. Additionally, morphological identification of worms can be done non-invasive (*i.e.*, individuals can be released following examination), provided the worms are not stored in preservative agents (*e.g.*, ethanol). Depending on the identification key as well as the required accuracy, even citizen science approaches are possible, enabling larger surveys at low costs. Such approaches, however, are often limited to identification at genus level or ecological/morphological groups (*e.g.*, Stroud, 2019; Billaud, Vermeersch & Porcher, 2021). Therefore, they are only suitable for certain research questions. Even if morphological identification is carried out by trained specialists, identification of juveniles or damaged tissue is often not possible, consequently leading to their exclusion in surveys (Richard et al., 2010).

DNA barcoding of taxonomically informative loci such as cytochrome c oxidase I (COI) genes is a popular molecular approach for the identification of metazoa (Hebert et al., 2003). COI is the dominating marker gene for metazoa and efforts were undertaken to build databases for COI sequencing data. For example, in 2005, The Barcode of Life Data Systems (BOLD) was launched to acquire, store, and analyze COI gene sequence data (Ratnasingham & Hebert, 2007). The potential of DNA barcoding for the identification of earthworms has been pointed out over a decade ago (Huang et al., 2007), but is still rarely used in practice (*e.g.*, Rutgers et al., 2016). Richard et al. (2010) highlighted that DNA barcoding enables identification of earthworms at all life stages, including juveniles, which has the potential to re-integrate juvenile individuals in earthworm studies and reduce possible identification bias.

High-resolution melting (HRM) curve analysis is a rapid and cost-effective tool, apart from the cost-intensive basic equipment, that allows differentiation among PCR products that differ by as little as one base pair. In this post-PCR technique, PCR products are differentiated by their thermal denaturation properties; their so-called “melting behaviour”. For this, the PCR product is heated stepwise (commonly 0.1 or 0.2 ° C per step) and the dissociation of the double-stranded DNA is quantified after every step using a fluorescent DNA-intercalating dye. Amplification and subsequent HRM curve analysis are commonly performed in a real-time PCR thermocycler. The main fields of application are genotyping and the detection of mutations. Analysis of HRM curves is successfully used in molecular diagnostics to distinguish clinically relevant bacteria (*e.g.*, Naze et al., 2015), fungi (*e.g.*, Fidler et al., 2016), and viruses (*e.g.*, Lin et al., 2008). Furthermore, HRM curve analysis can serve as an alternative tool to DNA barcoding for the taxonomic identification and differentiation of species (*e.g.*, Ngui, Lim & Chua, 2012). The analysis of HRM curves can also be used to identify invertebrates such as mosquitos (Ajamma et al., 2016), oysters (Wang et al., 2014), and nematodes (Skorpikova et al., 2020) as well as vertebrates (Ouso et al., 2020). Recently, the potential of HRM curve analysis for the differentiation of cryptic earthworm species was shown by Baudrin et al. (2020), who performed DNA barcoding and HRM analysis on a subregion of the 16S rRNA gene of the earthworm species *Allolobophora chlorotica*.

This work aimed to (i) demonstrate the potential of HRM curve analysis of the COI gene for the distinction of earthworm species and haplotypes common to agricultural soils in Central Europe and (ii) develop a rapid and inexpensive extraction method for DNA from earthworm tissue to reduce the costs for molecular analyses.

## Materials & Methods

### Reference earthworm material

Earthworm species common to agricultural soils in Central Europe were identified utilizing the Edaphobase database (Burkhardt et al., 2014). Eight agriculturally relevant species were selected (*Allolobophora chlorotica*, *Aporrectodea caliginosa*, *Apo. limicola*, *Apo. longa*, *Apo. rosea*, *Lumbricus castaneus*, *L. rubellus*, and *L. terrestris*) covering 87.6% of all database entries. Morphologically identified reference material of all eight species sampled in agricultural soils in Germany was kindly provided by Dr. Stefanie Krück. Total DNA was extracted using Qiagen’s DNeasy^®^ Blood & Tissue extraction kit (Qiagen N.V., Hilden, Germany) according to the manufacturers’ instructions. The extracts were checked on 1.7% (w/v) agarose gels stained with SYBR Green I. Prior to amplification, extracts were diluted 1:50 (v/v) in double-distilled H2O (ddH_2_O). For the identification of different haplotypes, DNA from 28 morphological identified individuals of *Allo. chlorotica* collected in October 2021 at an agricultural field near Otterndorf, Germany (53°48′32.69″N, 8°54′2.45″E) was extracted using a cetyltrimethylammonium bromide (CTAB)-based protocol as per Brandfass & Karlovsky (2008). Extracts were checked on 1.7% agarose gels and diluted 1:50 prior to PCR as described above.

### Rapid extraction method of DNA from earthworm tissue

We developed a rapid, robust, and inexpensive extraction method to extract amplifiable DNA from earthworm tissue. The protocol was tested using earthworm tissue of *Allo. chlorotica, Apo. caliginosa*, and *L. terrestris*. Fresh or frozen earthworm tissue (approx. 2 × 5 mm tissue) was placed in 1.5 mL tubes containing 50 µL ddH_2_O and two spatula tips of glass beads (Ø250–300 µm). The tissue was ground within the tube for 10 s using a sterile plastic micropestle and incubated at 70 °C for 10 min. Following incubation, the tube was centrifuged at 10,000× g for 1 min and the supernatant was transferred into a new 1.5 mL tube. The supernatant was diluted 1:10 (v/v) in ddH_2_O prior to PCR.

### Primer design for HRM curve analysis

We designed three forward and three reverse primers (Table 1) targeting different subregions of the COI gene. Reference sequences of all eight earthworm species were obtained from NCBI’s GenBank (accession numbers are given in Fig. S1) and aligned in MEGA version 11.0.10 (Kumar, Stecher & Tamura, 2016) using ClustalW (Thompson, Higgins & Gibson, 1994). Primers were designed manually by selecting suitable primer binding sites (Fig. S1 for primer pair EW_COI_F2 and EW_COI_R1, Fig. S2 for all primer) within the COI genes under consideration of potential dimerization, melting temperature, and degeneracy of the designed primers as well as sequence heterogeneity among species.

**Table 1:** Primer used for high-resolution melting (HRM) curve analysis.

**Primer name**	**Primer sequence (5′–3′)**	**Source**
EW_COI_F1	CATG CATT YGTD ATAA TYTT CTT	This study
EW_COI_F2	GTVT TYAT YGGN GGNT TYGG AAA	This study
EW_COI_F3	ATRG TDGG DGCH GGWA TRAG	This study
EW_COI_R1	CCDG THCC DGCN CCYT TTTC	This study
EW_COI_R2	AGAA TNAG NGAD GGRG GNAR NA	This study
EW_COI_R3	GADG CWCC HGCY ARRT GDAR DGA	This study
16S-Ac-F1	CTAAATTCTGACCCTTATTC	King et al. (2010)
WORM-16S-R1	CCTAAGCCAACATCGAGGTG	King et al. (2010)
COI-Al-F2	TGGCTTCTACCTCTAATACT	King et al. (2010)
COI-Al-R2	ATGAAGGGAGAAGATGGCCA	King et al. (2010)

### Development of HRM curve assay

Morphologically identified reference DNA material of all eight earthworm species was amplified using nine different primer combinations (Table 2). Amplifications were carried out in a CFX 384 Thermocycler (Biorad, Rüdigheim, Germany) in 384-well microplates in 4 µL reaction volumes consisting of ddH_2_O; buffer (10 mM Tris-HCl, 50 mM KCl, 2.5 mM MgCl_2_, pH 8.3 at 25 °C); 125 µM of each deoxynucleoside triphosphate (Bioline, Luckenwalde, Germany); 0.3 µM of each EW_COI primer (Table 1); 1 µg µL^−1^ bovine serum albumin; 0.5 µM EvaGreen^®^ solution (Jena Bioscience, Jena, Germany); 0.03 u µL^−1^ Hot Start Taq DNA Polymerase (New England Biolabs, Beverly, Massachusetts, USA) and 1 µL template DNA or ddH_2_O for negative controls. Thermocycling conditions for all nine primer combinations were as follows: Initial denaturation for 120 s at 95 °C followed by 40 cycles of denaturation for 20 s at 95 °C, annealing for 30 s at 55 °C, and elongation for 30 s at 68 °C. Final elongation was performed for 5 min at 68 °C. Following amplification, PCR products were heated to 95 °C for 60 s, cooled to 65 °C for 60 s, followed by a stepwise temperature increase from 65 °C to 95 °C by 0.1 °C per step with continuous fluorescence measurement to generate HRM curves.

**Table 2:** Primer combinations tested in this study and their PCR product length.

**Primer combination**	**PCR product length (bp)**
EW_COI_F1 × EW_COI_R1	194
EW_COI_F1 × EW_COI_R2	287
EW_COI_F1 × EW_COI_R3	152
EW_COI_F2 × EW_COI_R1	158
EW_COI_F2 × EW_COI_R2	251
EW_COI_F2 × EW_COI_R3	116
EW_COI_F3 × EW_COI_R1	293
EW_COI_F3 × EW_COI_R2	386
EW_COI_F3 × EW_COI_R3	251

Since a clear distinction between *Apo. caliginosa* and *Apo. longa* was not achieved by the optimized HRM curve assay (see *HRM curve analysis of earthworms*), we expanded a previously described multiplex PCR assay (King et al., 2010) for these two species by the generation of melting curves. Briefly, a 116 bp subregion of the 16S rRNA gene of *Apo. caliginosa* was amplified using the primer pair 16S-Ac-F1 and WORM-16S-R1 or a 213 bp subregion of the COI gene of *Apo. longa* using the primer pair COI-Al-F2 and COI-Al-R2 (Table 1). The composition of the reaction volume as well as the thermocycling conditions were identical to those described above except that four primers were used.

Furthermore, we aimed to test the suitability of our optimized HRM curve assay for the identification of different COI haplotypes. For this, HRM curve analysis was performed on different COI haplotypes across 28 individuals of *Allo. chlorotica* using the primer pair EW_COI_F2 and EW_COI_R1. The composition of the reaction volume as well as the thermocycling conditions were identical to those described for the HRM curve analysis of all eight earthworm species above. To confirm the findings from HRM curve analysis of potentially different haplotypes, COI genes of all 28 individuals of *Allo. chlorotica* were sequenced as described below (see *Sanger sequencing of the COI region of Allolobophora chlorotica*).

### Sanger sequencing of the COI region of Allolobophora chlorotica

The COI gene of 28 individuals of *Allo. chlorotica* was amplified using the primer set LCO1490 (GGTCAACAAATCATAAAGATATTGG) and HC02198 (TAAACTTCAGGGTGACCAAAAAATCA) (Folmer et al., 1994). Amplifications were carried out in an Eppendorf Mastercycler EP Gradient S thermocycler (Eppendorf, Hamburg, Germany) in 200 µL PCR tubes in 15 µL reaction volumes consisting of ddH_2_O; buffer (20 mM Tris-HCl, 10 mM (NH_4_)_2_SO_4_, 10 mM KCl, 2 mM MgSO_4_, 0.1% Triton^®^ X-100, pH 8.8 at 25 °C); 125 µM of each deoxynucleoside triphosphate (Bioline, Luckenwalde, Germany); 0.3 µM of each primer (LCO 1490 and HCO2198); 1 µg µL^−1^ bovine serum albumin; 0.03 u µL^−1^ Taq DNA Polymerase (New England Biolabs, Beverly, MA, USA) and 1 µL template DNA or ddH_2_O for negative controls. Thermocycling conditions were as follows. Initial denaturation for 120 s at 95 °C followed by 40 cycles of denaturation for 20 s at 95 °C, annealing for 30 s at 55 °C, and elongation for 60 s at 68 °C. Final elongation was performed for 5 min at 68 °C. Following amplification, PCR products were loaded on 1.7% agarose gels, bands of the expected product size were excised from the gel, and extracted utilizing the FastGene Gel/PCR Extraction Kit (Nippon Genetics Europe GmbH, Düren, Germany) according to the manufacturer’s instructions. Extracts were diluted 1:50 (v/v) in ddH_2_O and re-amplified as described above. Re-amplified PCR products were purified using isopropanol as described previously (Beule et al., 2017), quantified using a spectrophotometer (BioPhotometer plus, Eppendorf, Hamburg, Germany), and subjected to Sanger sequencing at the facilities of LGC Genomics (Berlin, Germany). The quality of the obtained sequences was checked manually and sequences were aligned in MEGA version 11.0.10 (Kumar, Stecher & Tamura, 2016) using ClustalW (Thompson, Higgins & Gibson, 1994). A phylogenetic tree was constructed using maximum likelihood analysis. All sequences were deposited at NCBI’s GenBank (accession numbers ON242065 to ON242092).

### HRM data processing and taxonomic assignment

HRM data was processed as described by Schiwek et al. (2020). Briefly, relative fluorescence unit (RFU) data was obtained from the CFX Maestro™ Software (Bio-Rad CFX Maestro 1.1 version 4.1.2433.1219, Biorad, Rüdigheim, Germany) and the negative first derivate was calculated to obtain melting curves. Difference curves were generated by subtracting the melting curve data of each reference earthworm from the mean melting curve data of all reference earthworms. All raw fluorescence and negative derivative of fluorescence data can be found in File S1.

## Results

### Rapid and inexpensive extraction of earthworm DNA

We developed a rapid extraction method to recover earthworm DNA from fresh or frozen tissue in less than 15 min. The method is easy, robust, and scalable. Furthermore, the method requires only minimal laboratory equipment (incubation at 70 °C and centrifugation) and comes at extremely low costs. As expected, extracted DNA was fragmented but sufficient amounts of amplifiable DNA were obtained (Fig. S3).

### HRM curve analysis of earthworms

**Figure 1:**
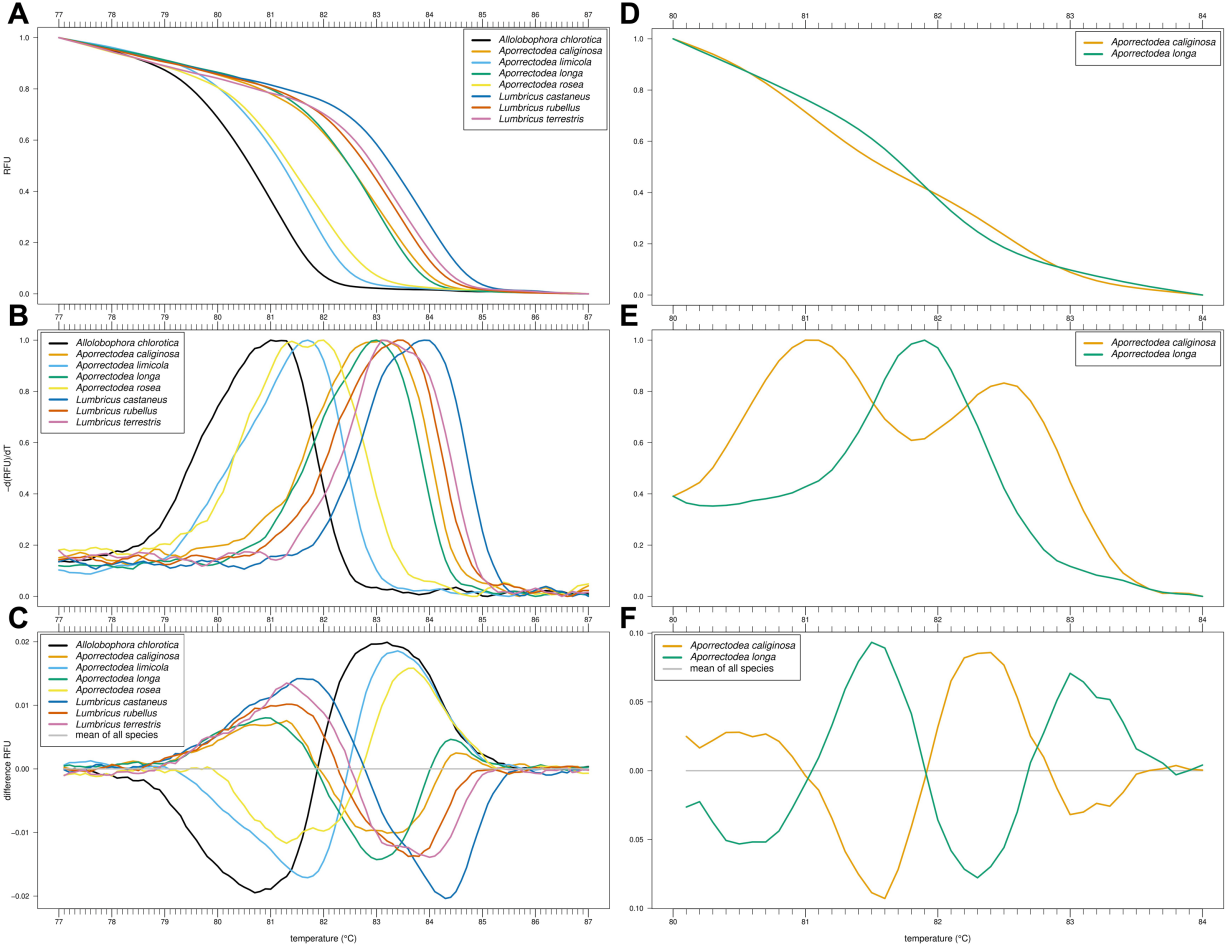
High-resolution melting (HRM) curve assay for the identification of earthworm species. Denaturation curves of eight earthworm species (A) and *Aporrectodea*. *caliginosa* and *Apo*. *longa* (D). Melting curves of eight earthworm species (B) and *Apo*. *caliginosa* and *Apo*. *longa* (E). Difference curves of eight earthworm species (C) and *Apo*. *caliginosa* and *Apo*. *longa* (F). PCRs were carried out using the primer pair EW_COI_F2 and EW_COI_R1. Differences curves were generated by subtracting the data of each curve from the mean of all curves. All curves represent means of five technical replicates.

Of our nine primer combinations, only the combination EW_COI_F2 and EW_COI_R1 (see Fig. S1 for alignment) yielded successful amplification of all eight species and enabled the distinction of *Allo. chlorotica*, *Apo. limicola*, *Apo. rosea*, *Lumbricus castaneus*, *L. rubellus*, and *L. terrestris* (Figs. 1A–1C). The subsequent multiplex real-time PCR assay with HRM curve analysis to distinguish *Allo*. *caliginosa* from *Allo*. *longa* was done using different markers (see *Development of HRM curve assay*) (Figs. 1D–1F).

**Figure 2:**
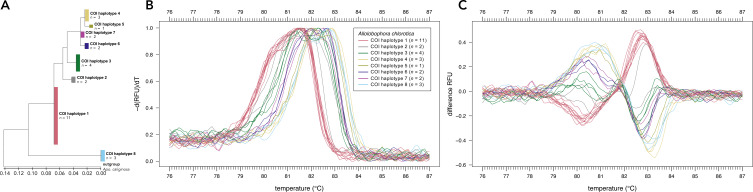
High-resolution melting (HRM) curve assay for the distinction of earthworm COI haplotypes of *Allolobophora chlorotica*. Phylogenetic tree of 28 individuals of *Allo. chlorotica* (A). The tree was constructed from the COI subregion amplified by the primer pair EW_COI_F2 and EW_COI_R1. The tree was constructed using maximum likelihood analysis. Melting curves (B) and difference curves (C) of 28 individuals of *Allo. chlorotica*. PCRs were carried out using the primer pair EW_COI_F2 and EW_COI_R1. Differences curves were generated by subtracting the data of each curve from the mean of all curves.

Sanger sequencing of the COI region of 28 individuals of *Allo. chlorotica* revealed eight different COI sequence variants (COI haplotypes) among individuals within the COI subregion amplified by the primer pair EW_COI_F2 and EW_COI_R1 (Fig. 2A). HRM curve analysis of the 28 individuals of *Allo. chlorotica* using the primer pair EW_COI_F2 and EW_COI_R1 mirrored the genetic distances among COI haplotypes (cf. Figs. 2A–2C). We were able to successfully distinguish several different COI haplotypes within *Allo. chlorotica* (Figs. 2B, 2C); however, the samples clustered into three groups of COI haplotypes. Group 1 of COI haplotypes comprised haplotype 1, group 2 comprised haplotypes 2, 3, 6, and 7, and group 3 comprised haplotypes 4, 5 and 8. None of the COI haplotypes of *Allo. chlorotica* reported in this work were identical with any previously reported COI haplotype.

## Discussion

Identification of soil fauna is an essential part of belowground biodiversity research and new primer sets targeting soil fauna are continuously being developed (*e.g.*, Capra et al., 2016). Most studies that used amplicon sequencing for metabarcoding of invertebrate communities worked with communities captured in traps and just few studies aimed to sequence soil fauna directly from soil samples (*e.g.*, Watts et al., 2019). Although direct amplicon sequencing from soil offers high taxonomic resolution as well as the ability to include invertebrates irrespective of their life stage and locomotion, such approaches have certain disadvantages. For example, amplicon sequencing data is compositional and does not reveal the absolute population size (Gloor et al., 2017) and thereby also ignores population dynamics (*e.g.*, Beule, Arndt & Karlovsky, 2021). Therefore, it was suggested to always accompany amplicon sequencing by absolute quantification approaches (Beule, Arndt & Karlovsky, 2021). Additionally, tremendous differences in the biomass of organisms lead to distinct differences in the DNA content per organism. Consequently, the number of sequences per individual is expected to increase with increasing biomass. Thus, relative abundances of taxa obtained from metabarcoding of complex communities reflect proportions of the biomass rather than population size. Earthworms are among the largest soil invertebrates and are frequently investigated in soil biodiversity surveys. For the sampling of earthworms, individuals are usually either extracted manually by hand-sorting or by using a combination of an expellant (*e.g.*, allyl isothiocyanate or formaldehyde) combined with hand-sorting. Such sampling and identification of individuals allows for the determination of population size and dynamics.

New tools for the identification of earthworms are continuously emerging. For example, in 2014, Fernández et al. (2014) showed that earthworms specimens can be identified taxonomically using micro-computed tomography. In 2021, the use of mid-infrared spectroscopy was proposed as a tool for earthworm identification (Pham et al., 2021). The same year, Andleeb et al. (2021) developed a machine-learning model for the identification of earthworms based on digital images. Currently, these techniques are rather experimental and have not established themselves in practice yet. A more widely established method besides traditional morphological identification is DNA barcoding of taxonomically informative loci such as COI.

Molecular tools such as DNA barcoding are associated with higher costs as morphological identification, partly due to the required basic equipment and the extraction of DNA that is commonly done using commercial extraction kits. Here, we introduce a simple, rapid, robust, inexpensive, and scalable homemade extraction method that enables the extraction of amplifiable DNA from earthworm tissue in less than 15 min. We believe that such techniques could help to promote the use of molecular techniques for earthworm taxonomy.

For large-scale use, HRM curve analysis is faster and less expensive compared to barcoding approaches as it is a closed-tube, post-PCR method. Still, investment costs for a real-time PCR thermocycler capable of generating HRM curves (*i.e.*, step-wise heating of PCR products at 0.1 to 0.2 °C per step under continuous fluorescence measurement) as well as costs for commercial software for the generation of HRM curves (unless raw fluorescence data is processed outside of a commercial software) remain a major hurdle for many laboratories. In the present study, we were able to distinguish eight earthworm species common to agricultural soils in Central Europe by using HRM curve analysis of a subregion of the COI gene (Fig. 1). However, as for barcoding approaches, suitable marker genes must be selected carefully. For example, large intraspecific variations in the COI gene of earthworms may limit its usage as a marker for DNA barcoding at species level (*e.g.*, Chang, Rougerie & Chen, 2009). Furthermore, since *Apo. caliginosa* and *Apo. longa* could not be clearly distinguished in the HRM curve assay alone, a subsequent multiplex PCR assay expanded by the generation of HRM curves must be performed to identify all eight species. Although our assay is an important first step towards HRM curve-based identification of earthworms, more work is required to overcome such limitations.

Sequencing of the COI gene of 28 individuals of *Allo. chlorotica* revealed eight different COI haplotypes within a short subregion (158 bp) of COI (Fig. 2A). Some of these COI haplotypes were distinguishable by using HRM curve analysis (Figs. 2B, 2C), highlighting its potential for intraspecies differentiation. However, we also found that several COI haplotypes (*i.e.*, those within COI haplotype group 2 and 3) are undistinguishable using our HRM curve assay, limiting haplotype typing. Although sequencing remains necessary in our case to uncover the full diversity of haplotypes, HRM curve-based differentiation of haplotypes can serve as a first indication of haplotype diversity. Similar findings among individuals of *Allo. chlorotica* were obtained by Baudrin et al. (2020) for a subregion of the 16S rRNA gene. Besides its potential to assess intraspecific variability, intraspecific marker gene variations may impede robust identification at species level using HRM curve analysis, particularly if the variation of melting profiles induced by intraspecific marker gene variation is large. Until suitable marker genes or marker gene subregions are found, we suggest that HRM curve analysis of earthworms could support morphological investigations, especially for juveniles and damaged specimens that cannot be identified morphologically. If combined, morphological identification and HRM curve analysis may be a suitable alternative to DNA barcoding.

## Conclusion

HRM curve analysis of COI genes has the potential to simultaneously identify earthworm species and assess intraspecific variations. For robust species identification, we encourage researchers to search for marker genes or marker gene subregions that are suitable for species differentiation by HRM curve analysis and have no to minimal intraspecific variation. Morphological identification remains the most common method to identify earthworm species; therefore, we suggest that HRM curve analysis can be used to confirm morphological identification, especially for juveniles and damaged tissue. Molecular analyses have several advantages over morphological identification but are cost and labour intensive. Our rapid and inexpensive DNA extraction method from earthworm tissue helps to reduce the costs of molecular analyses and thereby promote their application in practice.

## Supplemental Information

10.7717/peerj.13661/supp-1 Supplemental Information 1 Sequence alignment of the newly developed COI primer pair EW_COI_F2 and EW_COI_R1 used for identification of eight earthworm species by high-resolution melting curve analysis Reference sequences were obtained from NCBI’s GenBank (accession numbers are given in the figure) and aligned in MEGA version 11.0.10 (Kumar, Stecher & Tamura, 2016) using ClustalW (Thompson, Higgins & Gibson, 1994). For the reverse primer EW_COI_R1, the reverse complement is shown.

10.7717/peerj.13661/supp-2 Supplemental Information 2 Sequence alignment of the three newly developed COI primer pairs used for identification of eight earthworm species by high-resolution melting curve analysis Reference sequences were obtained from NCBI’s GenBank (accession numbers are given in the figure) and aligned in MEGA version 11.0.10 (Kumar, Stecher & Tamura, 2016) using ClustalW (Thompson, Higgins & Gibson, 1994). For reverse primers, the reverse complements are shown.

10.7717/peerj.13661/supp-3 Supplemental Information 3 Agarose gel (1% (w/v)) showing earthworm DNA extracts obtained by using the developed rapid and inexpensive extraction protocol Lanes 8 to 15: 3 µL of DNA extract of Allolobophora chlorotica, Aporrectodea caliginosa, Apo. limicola, Apo. longa, Apo. rosea, Lumbricus castaneus, L. rubellus, and L. terrestris, respectively. Lanes 6 and 17: 1 µL of 1 kb Plus DNA Ladder (New England Biolabs, Beverly, Massachusetts, USA). The agarose gel was stained using PicoGreen (Thermo Fisher Scientific, Waltham, MA, USA) and run at 4.6 V/cm for 60 min.

10.7717/peerj.13661/supp-4 Supplemental Information 4 Raw fluorescence and negative derivative of fluorescence data

## Additional Information and Declarations

### DNA Deposition

The following information was supplied regarding the deposition of DNA sequences: All sequences are available at NCBI’s GenBank: ON242065 to ON242092.

### Data Availability

The following information was supplied regarding data availability: All raw fluorescence and negative derivative of fluorescence data are available in the Supplementary File.
