# Supplementary figures and images for: High-resolution melting (HRM) curve analysis as a potential tool for the identification of earthworm species and haplotypes
Source: PeerJ. 2022 Jun 28;10:e13661. doi: 10.7717/peerj.13661 (PMC9248783; doi:10.7717/peerj.13661)

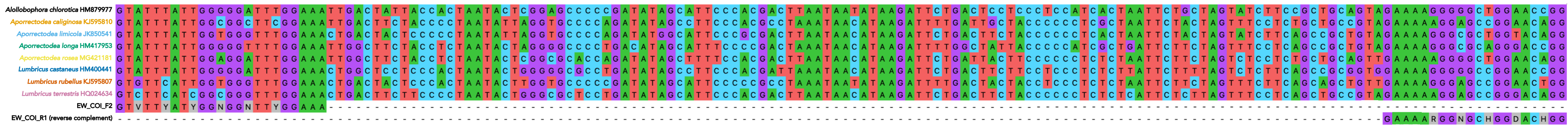

Supplement: Supplemental Information 1 — Reference sequences were obtained from NCBI’s GenBank (accession numbers are given in the figure) and aligned in MEGA version 11.0.10 (Kumar, Stecher & Tamura, 2016) using ClustalW (Thompson, Higgins & Gibson, 1994). For the reverse primer EW_COI_R1, the reverse complement is shown. [file peerj-10-13661-s001.pdf]

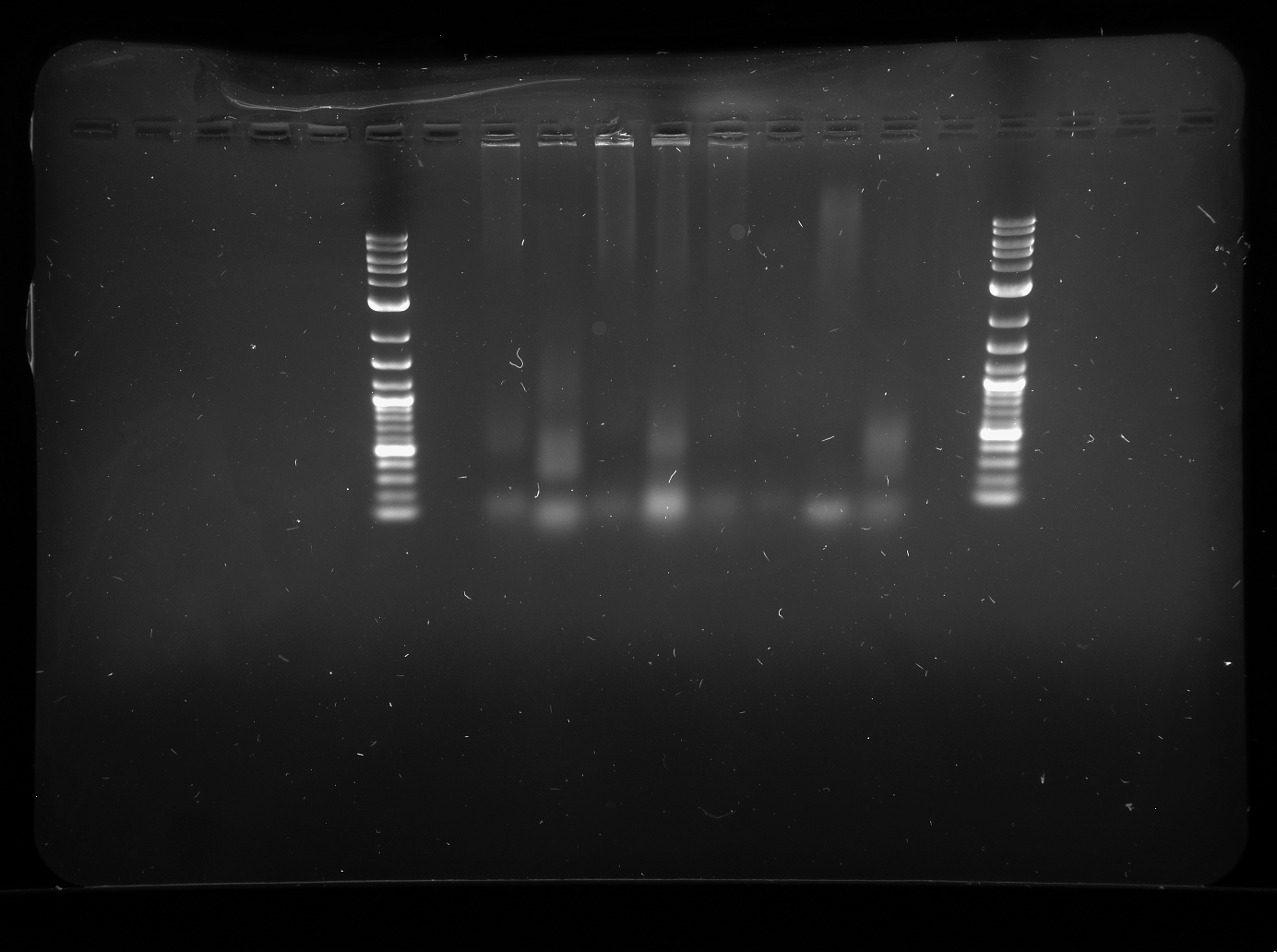

Supplement: Supplemental Information 3 — Lanes 8 to 15: 3 µL of DNA extract of Allolobophora chlorotica, Aporrectodea caliginosa, Apo. limicola, Apo. longa, Apo. rosea, Lumbricus castaneus, L. rubellus, and L. terrestris, respectively. Lanes 6 and 17: 1 µL of 1 kb Plus DNA Ladder (New England Biolabs, Beverly, Massachusetts, USA). The agarose gel was stained using PicoGreen (Thermo Fisher Scientific, Waltham, MA, USA) and run at 4.6 V/cm for 60 min. [file peerj-10-13661-s003.tif]
