# Supplementary material for: High-resolution melting (HRM) curve analysis as a potential tool for the identification of earthworm species and haplotypes
Source: PeerJ. 2022 Jun 28;10:e13661. doi: 10.7717/peerj.13661 (PMC9248783; doi:10.7717/peerj.13661)
Supplement: Supplemental Information 2 — Reference sequences were obtained from NCBI’s GenBank (accession numbers are given in the figure) and aligned in MEGA version 11.0.10 (Kumar, Stecher & Tamura, 2016) using ClustalW (Thompson, Higgins & Gibson, 1994). For reverse primers, the reverse complements are shown. [file peerj-10-13661-s002.pdf]

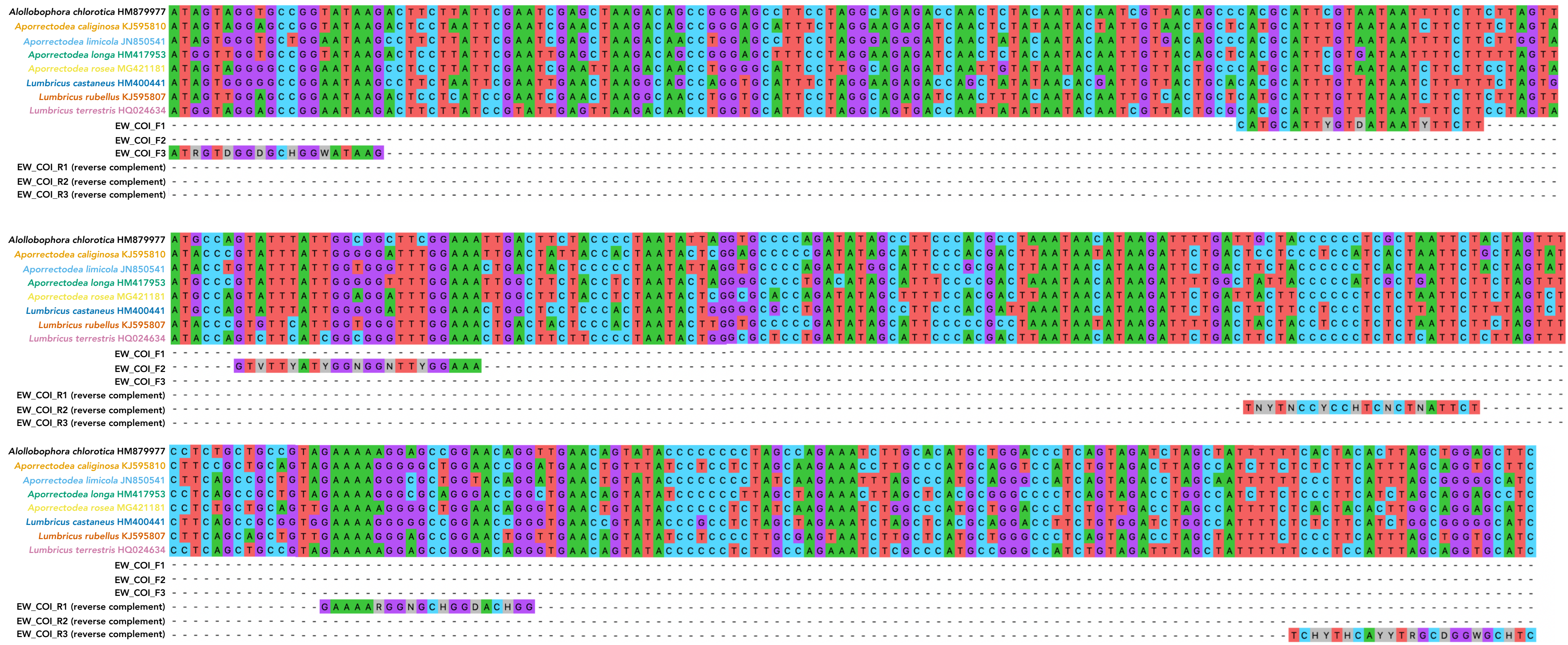
Alollobophora chlorotica HM879977
Aporrectodea caliginosa KJ595810
Aporrectodea limicola JN850541
Aporrectodea longa HM417953
Aporrectodea rosea MG421181
Lumbricus castaneus HM400441
Lumbricus rubellus KJ595807
Lumbricus terrestris HQ024634
EW_COI_F1 CATGCATTYGTDATAATYTTCTT
EW_COI_F2 GTVTTYATYGGNGGNTTYGGAAA
EW_COI_F3 ATRGTDGGDGCHGGWATAAG
EW_COI_R1 (reverse complement) GAAAARGGNGCHGGDACHGG
EW_COI_R2 (reverse complement) TNYTNCCYCCHTCNCTNATTCT
EW_COI_R3 (reverse complement) TCHYTHCAYYTRGCDGGWGCHTC
